# In Utero Nutritional Manipulation Provokes Dysregulated Adipocytokines Production in F1 Offspring in Rats

**DOI:** 10.1155/2016/3892890

**Published:** 2016-04-20

**Authors:** Mervat Y. Hanafi, Mohamed I. Saad, Taha M. Abdelkhalek, Moustafa M. Saleh, Maher A. Kamel

**Affiliations:** ^1^Department of Biochemistry, Medical Research Institute, Alexandria University, Alexandria, Egypt; ^2^The Ritchie Centre, Hudson Institute of Medical Research, Monash University, Melbourne, VIC, Australia; ^3^Department of Human Genetics, Medical Research Institute, Alexandria University, Alexandria, Egypt

## Abstract

*Background*. Intrauterine environment plays a pivotal role in the origin of fatal diseases such as diabetes. Diabetes and obesity are associated with low-grade inflammatory state and dysregulated adipokines production. This study aims to investigate the effect of maternal obesity and malnutrition on adipokines production (adiponectin, leptin, and TNF-*α*) in F1 offspring in rats.* Materials and Methods*. Wistar rats were allocated in groups: F1 offspring of control mothers under control diet (CF1-CD) and under high-fat diet (CF1-HCD), F1 offspring of obese mothers under CD (OF1-CD) and under HCD (OF1-HCD), and F1 offspring of malnourished mothers under CD (MF1-CD) and under HCD (MF1-HCD). Every 5 weeks postnatally, blood samples were obtained for biochemical analysis.* Results*. At the end of the 30-week follow-up, OF1-HCD and MF1-HCD exhibited hyperinsulinemia, moderate dyslipidemia, insulin resistance, and impaired glucose homeostasis compared to CF1-CD and CF1-HCD. OF1-HCD and MF1-HCD demonstrated low serum levels of adiponectin and high levels of leptin compared to CF1-CD and CF1-HCD. OF1-CD, OF1-HCD, and MF1-HCD had elevated serum levels of TNF-*α* compared to CF1-CD and CF1-HCD (*p* < 0.05).* Conclusion*. Maternal nutritional manipulation predisposes the offspring to development of insulin resistance in their adult life, probably via instigating dysregulated adipokines production.

## 1. Introduction

Increasing evidence from clinical and epidemiological research suggests that prenatal environment plays a crucial role in the origin of fatal diseases such as the metabolic syndrome and its components: insulin resistance, hypertension, and dyslipidemia [1]. Maternal nutritional environment plays rather pivotal role in the programming of the health and disease of the offspring during the adult life. In particular, maternal obesity is associated with increased prevalence of cardiovascular diseases in the offspring [2]. This intrauterine programming might develop at the gene, cellular, tissue, organ, and system levels, resulting in long-lasting structural and functional modifications [3].

Adiponectin is an insulin-sensitizing, antidiabetic, antioxidant, anti-inflammatory, and antiatherogenic adipokine, which acts on adiponectin receptors, for example, AdipoR1 and AdipoR2 to exert its actions [4]. Leptin is another adipokine involved in the pathogenesis of obesity, insulin resistance, inflammation, and diabetes. It exerts a regulatory action on satiety and energy homeostasis, production of inflammatory mediators, and lipid and carbohydrate metabolism [4]. Diabetes, metabolic syndrome, and obesity are associated with decreased levels of adiponectin and elevated levels of leptin. This state of adiponectin deficiency and leptin resistance could be a possible mechanism for development of diabetes and its complications [4–6].

Subclinical inflammation is a hallmark of obesity, diabetes, and metabolic syndrome [4]. Tumor necrosis factor-*α* (TNF-*α*) is an inflammatory cytokine that has a variety of functions such as mediating apoptosis and regulation of immune system. TNF-*α* exerts its effects via its action on TNFR1 and TNFR2 receptors [4]. Serum TNF-*α* level is elevated in obese and diabetic patients and is correlated with various complications of diabetes [7, 8].

Epigenetic modifications, deterioration of glucose tolerance, elevated inflammatory markers, and excessive oxidative stress could be possible mechanisms for the intrauterine programming of fetal diseases [2, 9, 10]; however, the exact mechanism is not fully understood. We have shown that maternal obesity and undernutrition affect glucose sensing and mitochondrial functions in the offspring [11]. The aim of this study is to investigate the effect of maternal nutritional manipulation on adipocytokines production (adiponectin, leptin, and TNF-*α*) in F1 offspring in rats, as a potential mechanism for the intergenerational effect of maternal nutritional environment on the offspring.

## 2. Materials and Methods

### 2.1. Animals

The study was done in accordance with the ethical guidelines of the Medical Research Institute, Alexandria University, Alexandria, Egypt. Wistar rats were housed as 4 per cage at an ambient temperature of 23 ± 1°C with 12/12 h light/dark cycles and 45 ± 5% humidity.

### 2.2. Experimental Design

Female rats were allocated randomly in three groups: control, obese, and malnourished. Obesity was instigated via maintaining female neonates under obesogenic diet for two months after weaning. Female rats that were 20% heavier than controls of the same age were considered obese. Malnutrition was instigated through maintaining female neonates under low-protein diet (8% protein) for two months after weaning. Female rats that were 20% lighter than controls of the same age were considered malnourished.

Pregnancy was carried out by overnight mating the females (control, obese, and malnourished) with normal healthy males. Pregnancy was confirmed next morning by the presence of vaginal mucus plug. Pregnancies were completed to term. After delivery, the offspring were weaned to control diet (CD) or high-caloric diet (HCD) and followed up for 30 weeks. Therefore, the offspring groups were as follows: F1 offspring of control mothers under CD (CF1-CD), F1 offspring of control mothers under HCD (CF1-HCD), F1 offspring of obese mothers under CD (OF1-CD), F1 offspring of obese mothers under HCD (OF1-HCD), F1 offspring of malnourished mothers under CD (MF1-CD), and F1 offspring of malnourished mothers under HCD (MF1-HCD). Pregnancy outcome and composition of diets used were previously published [12]. Every 5 weeks postnatally, 10 pups of each subgroup were culled after overnight fasting to obtain blood samples for biochemical analysis.

### 2.3. Biochemical Analysis

Fasting blood glucose (FBG) level was measured using glucometer (OneTouch, Johnson & Johnson Co.). A commercial diagnostic kit (Randox (UK)) was used to assess lipid profile according to the manufacturer's instructions.

### 2.4. ELISA Measurements

Plasma insulin was assessed using ELISA kit (Mercodia). Serum levels of adiponectin, leptin, NEFA, and TNF-*α* were assessed using ELISA kits (Chemicon, RayBio, MyBioSource, and R&D Systems, resp.) according to the manufacturer's instructions.

### 2.5. Statistical Analysis

All statistical analyses were performed using SPSS statistical software version 18 (SPSS, Chicago, IL). The data were analyzed using the one-way analysis of variance (ANOVA) followed by LSD test to compare the mean values from the offspring of obese and malnourished mothers and the offspring of control mothers.* t*-test was employed to compare the mean values of females and those of males of the same group at the same age. The results are presented as mean ± SD. Values of *p* > 0.05 were considered nonsignificantly different, while those of *p* < 0.05 were considered significant.

## 3. Results

### 3.1. Glucose Homeostasis Parameters

#### 3.1.1. Fasting Blood Glucose (FBG) Level

**Figure 1:**
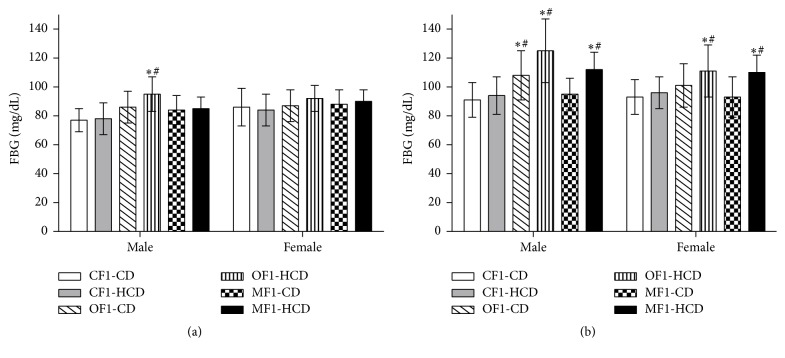
Fasting blood glucose (FBG) levels (mg/dL) of all study groups at (a) week 5 and (b) week 30 of age. Data are presented as mean ± SD (*n* = 10). CF1-CD: F1 offspring of control mothers under control diet, CF1-HCD: F1 offspring of control mothers under high-caloric diet, OF1-CD: F1 offspring of obese mothers under control diet, and OF1-HCD: F1 offspring of obese mothers under high-caloric diet; MF1-CD: F1 offspring of malnourished mothers under control diet and MF1-HCD: F1 offspring of malnourished mothers under high-caloric diet. The symbol *∗* indicates significant difference from CF1-CD by ANOVA (*p* < 0.05) and the symbol # indicates significant difference from CF-HCD by ANOVA (*p* < 0.05).

At week 5, only males of OF1-HCD showed significantly higher FBG level than CF1-CD and CF1-HCD groups. At week 30, males and females of OF1-HCD and MF1-HCD exhibited significantly higher FBG levels than CF1-CD and CF1-HCD groups. Furthermore, males of OF1-CD had significantly higher FBG level compared to CF1-CD and CF1-HCD groups at the 30th week (Figure 1).

#### 3.1.2. Serum Insulin Level

**Figure 2:**
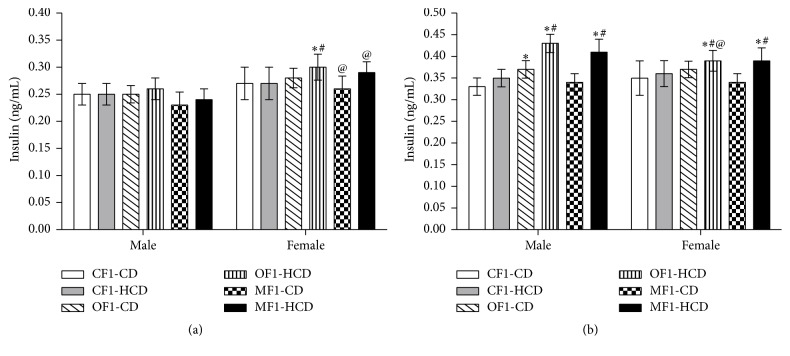
Serum insulin levels (ng/mL) of all study groups at (a) week 5 and (b) week 30 of age. Data are presented as mean ± SD (*n* = 10). CF1-CD: F1 offspring of control mothers under control diet, CF1-HCD: F1 offspring of control mothers under high-caloric diet, OF1-CD: F1 offspring of obese mothers under control diet, and OF1-HCD: F1 offspring of obese mothers under high-caloric diet; MF1-CD: F1 offspring of malnourished mothers under control diet and MF1-HCD: F1 offspring of malnourished mothers under high-caloric diet. The symbol *∗* indicates significant difference from CF1-CD by ANOVA (*p* < 0.05), the symbol # indicates significant difference from CF-HCD by ANOVA (*p* < 0.05), and the symbol @ indicates significant difference from male at each age by *t*-test.

At week 5, only females of OF1-HCD showed significantly higher insulin levels than CF1-CD and CF1-HCD groups. Also, females of MF1-CD and MF1-HCD groups showed higher insulin levels than their male counterparts (Figure 2). At week 30, males of OF1-CD group as well as all members of OF1-HCD and MF1-HCD groups exhibited significantly higher insulin levels than CF1-CD and CF1-HCD group. Only males of OF1-HCD had significant higher insulin level compared to their female counterparts (Figure 2).

#### 3.1.3. Homeostasis Model Assessment of Insulin Resistance (HOMA-IR)

**Figure 3:**
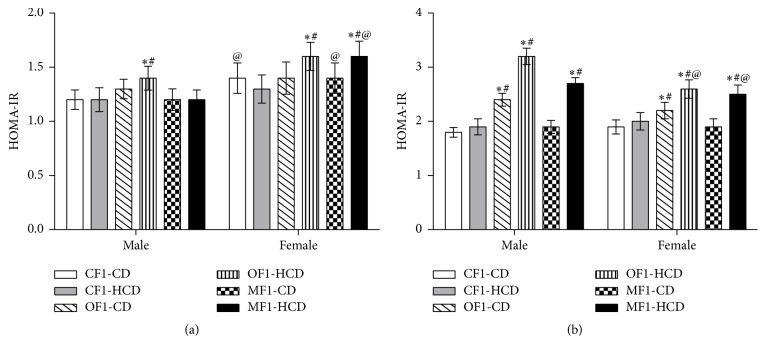
Homeostasis model assessment of insulin resistance (HOMA-IR) of all study groups at (a) week 5 and (b) week 30 of age. Data are presented as mean ± SD (*n* = 10). CF1-CD: F1 offspring of control mothers under control diet, CF1-HCD: F1 offspring of control mothers under high-caloric diet, OF1-CD: F1 offspring of obese mothers under control diet, and OF1-HCD: F1 offspring of obese mothers under high-caloric diet; MF1-CD: F1 offspring of malnourished mothers under control diet and MF1-HCD: F1 offspring of malnourished mothers under high-caloric diet. The symbol *∗* indicates significant difference from CF1-CD by ANOVA (*p* < 0.05), the symbol # indicates significant difference from CF-HCD by ANOVA (*p* < 0.05), and the symbol @ indicates significant difference from male at each age by *t*-test.

The insulin resistance index calculated by the HOMA model (HOMA-IR) using fasting serum levels of insulin (*μ*IU/mL) and glucose levels (mmol/L) indicated that males and females of OF1-HCD group as well as females of MF1-HCD were insulin resistant compared to CF1-CD and CF1-HCD groups at the 5th week (Figure 3). At the 30th week, all members of OF1-CD, OF1-HCD, and MF1-HCD groups were insulin resistant compared to CF1-CD and CF1-HCD groups (Figure 3).

### 3.2. Lipid Profile

**Figure 4:**
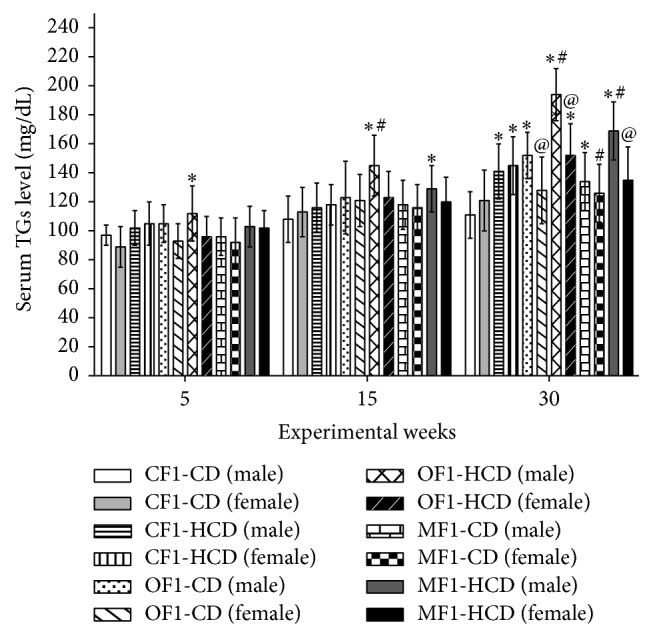
Serum levels of triglycerides (TGs) (mg/dL) of all study groups. Data are presented as mean ± SD (*n* = 10). CF1-CD: F1 offspring of control mothers under control diet, CF1-HCD: F1 offspring of control mothers under high-caloric diet, OF1-CD: F1 offspring of obese mothers under control diet, and OF1-HCD: F1 offspring of obese mothers under high-caloric diet; MF1-CD: F1 offspring of malnourished mothers under control diet and MF1-HCD: F1 offspring of malnourished mothers under high-caloric diet. The symbol *∗* indicates significant difference from CF1-CD by ANOVA (*p* < 0.05), the symbol # indicates significant difference from CF-HCD by ANOVA (*p* < 0.05), and the symbol @ indicates significant difference from male at each age by *t*-test.

At week 5, only males of OF1-HCD showed significantly higher serum triglycerides (TGs) level compared to CF1-CD. At week 15, males of OF1-HCD showed significantly higher TGs level compared to CF1-CD and CF1-HCD. Also, males of MF1-HCD exhibited significantly higher TGs level compared to CF1-CD. At the 30th week, all members of CF1-HCD and OF1-HCD as well as males of OF1-CD, MF1-CD, and MF1-HCD exhibited elevated TGs level compared to CF1-CD. Moreover, males of OF1-HCD showed elevated TGs level compared to CF1-HCD (Figure 4).

Members of OF1-HCD showed significantly higher hepatic TGs content compared to CF1-CD from the 10th week to the 30th week, except males at the 10th week. Also, OF1-HCD showed significantly higher hepatic TGs content compared to CF1-HCD from the 10th week to the 30th week, except males at weeks 10 and 15. Members of OF1-CD as well as females of MF1-HCD exhibited significant rise in TGs content in the liver compared to CF1-CD at only the 30th week (Table 1).

Members of OF1-CD showed significantly higher muscular TGs content compared to CF1-CD at weeks 25 and 30. Also, females of this group had higher muscular TGs content compared to CF1-HCD at the last two time points. OF1-HCD group (except males at the 5th week) showed significantly higher muscular TGs content compared to CF1-CD throughout the follow-up. Moreover, this group had significant rise in their muscular TGs content compared to CF1-HCD from weeks 10 and 30, except females at the 10th week. Members of MF1-CD and MF1-HCD demonstrated significantly elevated muscular TGs content compared to CF1-CD at the 30th week (Table 1).

**Figure 5:**
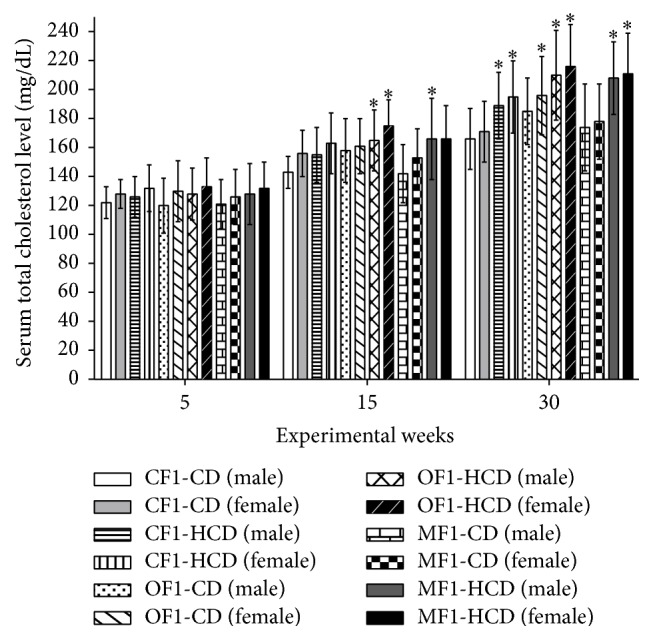
Serum levels of total cholesterol (mg/dL) of all study groups. Data are presented as mean ± SD (*n* = 10). CF1-CD: F1 offspring of control mothers under control diet, CF1-HCD: F1 offspring of control mothers under high-caloric diet, OF1-CD: F1 offspring of obese mothers under control diet, and OF1-HCD: F1 offspring of obese mothers under high-caloric diet; MF1-CD: F1 offspring of malnourished mothers under control diet and MF1-HCD: F1 offspring of malnourished mothers under high-caloric diet. The symbol *∗* indicates significant difference from CF1-CD by ANOVA (*p* < 0.05).

At week 15, members of OF1-HCD and males of MF1-HCD showed significantly elevated total serum cholesterol level compared to CF1-CD. At week 30, members of CF1-HCD, OF1-HCD, and MF1-HCD as well as females of OF1-CD exhibited significantly elevated total serum cholesterol level compared to CF1-CD (Figure 5).

**Figure 6:**
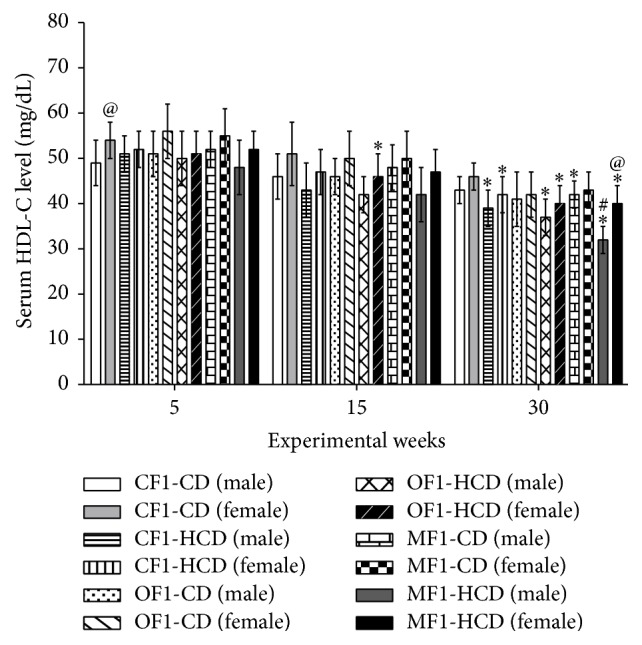
Serum levels of high-density lipoprotein-cholesterol (HDL-C) (mg/dL) of all study groups. Data are presented as mean ± SD (*n* = 10). CF1-CD: F1 offspring of control mothers under control diet, CF1-HCD: F1 offspring of control mothers under high-caloric diet, OF1-CD: F1 offspring of obese mothers under control diet, and OF1-HCD: F1 offspring of obese mothers under high-caloric diet; MF1-CD: F1 offspring of malnourished mothers under control diet and MF1-HCD: F1 offspring of malnourished mothers under high-caloric diet. The symbol *∗* indicates significant difference from CF1-CD by ANOVA (*p* < 0.05), the symbol # indicates significant difference from CF-HCD by ANOVA (*p* < 0.05), and the symbol @ indicates significant difference from male at each age by *t*-test.

At week 15, only females of OF1-HCD showed significantly lower serum high-density lipoprotein-cholesterol (HDL-C) levels than CF1-CD group. All members of CF1-HCD, OF1-HCD, and MF1-HCD as well as males of MF1-CD exhibited significantly lower serum HDL-C levels than CF1-CD group at the end of the follow-up period. Moreover, males of MF1-HCD demonstrated significant drop of their HDL-C levels compared to their female counterparts and CF1-HCD group (Figure 6).

**Figure 7:**
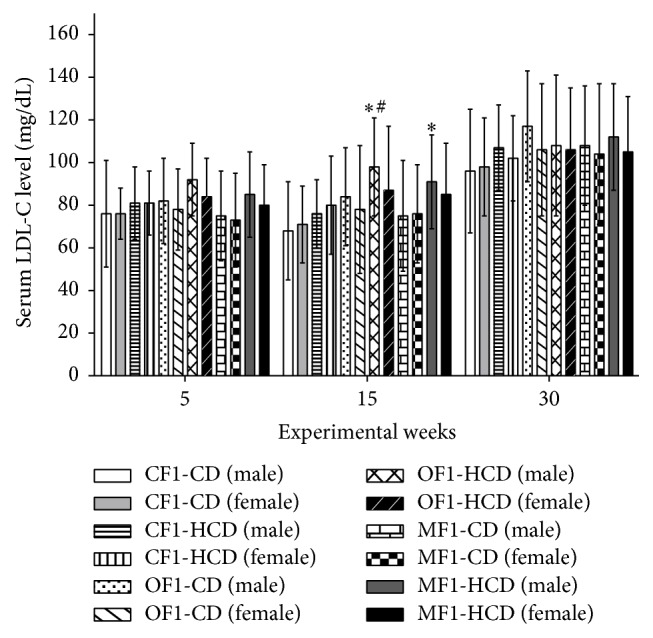
Serum levels of low-density lipoprotein-cholesterol (LDL-C) (mg/dL) of all study groups. Data are presented as mean ± SD (*n* = 10). CF1-CD: F1 offspring of control mothers under control diet, CF1-HCD: F1 offspring of control mothers under high-caloric diet, OF1-CD: F1 offspring of obese mothers under control diet, and OF1-HCD: F1 offspring of obese mothers under high-caloric diet; MF1-CD: F1 offspring of malnourished mothers under control diet and MF1-HCD: F1 offspring of malnourished mothers under high-caloric diet. The symbol *∗* indicates significant difference from CF1-CD by ANOVA (*p* < 0.05) and the symbol # indicates significant difference from CF-HCD by ANOVA (*p* < 0.05).

Regarding levels of low-density lipoprotein-cholesterol (LDL-C) levels, only males of OF1-HCD and MF1-HCD had significantly higher LDL-C levels than CF1-CD at the 15th week. Also, males of OF1-HCD showed significantly higher LDL-C levels than CF1-HCD at the 15th week (Figure 7).

All members of CF1-HCD group had a significant rise in their serum nonesterified fatty acids (NEFA) levels from week 10 to week 30 compared to CF1-CD, except females at weeks 15, 25, and 30 that did not show such significant difference. Also, males of OF1-CD showed significantly higher NEFA levels at weeks 10, 20, 25, and 30 compared to CF1-CD. All members of OF1-HCD (except females at weeks 5 and 30) showed higher NEFA levels compared to CF1-CD. Moreover, males of MF1-HCD from week 10 to week 30 and females of MF1-HCD at the 30th week demonstrated higher NEFA levels compared to CF1-CD (Table 2).

### 3.3. Serum Levels of Cytokines

Females had significantly higher serum adiponectin levels than their male counterparts in all study groups throughout the study period. Males of CF1-HCD and OF1-CD showed significantly lower serum adiponectin levels than CF1-CD from week 20 to week 30. All members of OF1-HCD (except males at week 5) showed significantly lower serum adiponectin levels than CF1-CD throughout the study period. Also, members of OF1-HCD had a significant drop in their serum adiponectin levels compared to CF1-HCD at weeks 25 and 30. Members of MF1-HCD (at weeks 20, 25, and 30) had significantly lower adiponectin levels compared to CF1-CD. Furthermore, females of MF1-HCD (at weeks 25 and 30) showed a significant drop in their adiponectin levels compared to CF1-HCD (Table 3).

Females had significantly elevated serum leptin levels than their male counterparts in all the study groups throughout the study period. Throughout the study, males of CF1-HCD (except at the 5th week), members of OF1-CD (except males at week 5), and all members of MF1-HCD (except males at week 5) had significantly higher leptin levels compared to CF1-CD. Moreover, females of OF1-CD (from week 10 to week 30), males of OF1-CD (at weeks 15 and 30), females of OF1-HCD (at all follow-up time points), males of OF1-HCD (at weeks 15 and 20), and females of MF1-HCD (from week 10 to week 30) demonstrated higher leptin levels compared to CF1-CD (Table 3).

Females had significantly higher serum TNF-*α* levels than their male counterparts in all the study groups (except CF1-HCD and OF1-CD at the 5th week) throughout the study period. All members of CF1-HCD (except females at the 5th, 10th, and 30th weeks), all members of OF1-CD (except males at week 15), all members of OF1-HCD, and all members of MF1-HCD (except males at the 5th week) showed significantly higher serum TNF-*α* levels compared to CF1-CD throughout the study period. Moreover, members of MF1-CD demonstrated significantly higher serum TNF-*α* levels compared to CF1-CD from week 20 to week 30. All members of OF1-CD from week 10 to week 30 (except males at week 15) showed significantly higher serum TNF-*α* levels compared to CF1-HCD. Furthermore, all members of OF1-HCD (except males at the 5th week) and MF1-HCD showed significantly higher serum TNF-*α* levels compared to CF1-HCD throughout the follow-up period (Table 3).

## 4. Discussion

According to the hypothesis of fetal origin of adult diseases, the intrauterine environment may have a critical impact on long-term health and disease of the offspring. Therefore, maternal intrauterine milieu may alter the metabolic status of the fetus, instigating an insulin resistance state and enhancing the development of type 2 diabetes mellitus and metabolic syndrome in the adult life, especially following postnatal obesogenic environment [13]. In support of this idea, our findings indicate that F1 offspring of obese and malnourished mothers exhibited impaired glucose homeostasis, moderate dyslipidemia, insulin resistance, and dysregulated adipokines production, predisposing the offspring for the development of diabetes and its complications, especially when they encounter diabetogenic environment in their adult life.

Dyslipidemia is an important risk factor for the development of cardiovascular diseases in the context of obesity and diabetes. The cardinal features of dyslipidemia are high plasma TGs level, low HDL-C level, and elevated level of small dense LDL-C particles. These lipid changes are attributed to the increased NEFA flux owing to adipose tissue insulin resistance [14]. The increase in NEFA flux induces insulin resistance in liver and skeletal muscles through direct or indirect (via triglyceride deposits) generation of metabolites and interfering with insulin signalling pathways [15]. Moreover, NEFA play a crucial role in *β*-cell dysfunction [16].

Hyperinsulinemia is a key player in the development of hepatosteatosis and hepatic insulin resistance [17]. Consistently, offspring of obese and malnourished mothers showed significant elevated TGs content in their livers and muscles.

Our data indicates that in utero maternal nutritional manipulation is sufficient for induction of dysregulated adipocytokines production in the offspring during their adult life, and the diabetogenic environment exaggerates this effect. Offspring of obese and malnourished mothers showed low levels of adiponectin, high levels of leptin, and elevated levels of TNF-*α*. Consistently, offspring of high-fat diet fed dams showed hyperinsulinemia, hyperleptinemia, decreased adiponectin levels, increased pancreatic mass, and islet volume density with elevated *α*- and *β*-cell mass [18]. Moreover, offspring from malnourished dams showed altered adipose tissue functions such as impaired glucose uptake, insulin and leptin resistance, low-grade inflammation, and modified sympathetic activity with reduced noradrenergic innervations [19].

There is a strong association between markers of inflammatory activity and endothelial dysfunction, the trigger for cardiovascular diseases [4]. Interestingly, increased inflammatory mediators may predict the future development of obesity and diabetes. The increased concentrations of TNF-*α* and interleukin-6 (IL-6) might interfere with insulin action by suppressing insulin signal transduction pathways [20].

In conclusion, maternal nutritional manipulation may predispose the offspring to development of insulin resistance in their adult life, probably via provoking a state of dysregulated adipocytokines production.

## Figures and Tables

**Table 1:** Hepatic and muscular content of TGs (mg/g tissue) in different study groups.

Age (weeks)	Sex	CF1-CD	CF1-HCD	OF1-CD	OF1-HCD	MF1-CD	MF1-HCD
*Hepatic TGs content (mg/g tissue)*							
5	Male	76 ± 7	79 ± 8	75 ± 7	81 ± 8	74 ± 8	77 ± 8
	Female	75 ± 6	76 ± 5	75 ± 6	77 ± 5	71 ± 6^#^	76 ± 6
10	Male	82 ± 8	84 ± 8	82 ± 7	88^*∗*^ ± 8	79 ± 7	82 ± 9
	Female	78 ± 5	81 ± 6	82 ± 7	86 ± 7^*∗*#^	75 ± 5	83 ± 8
15	Male	83 ± 7	92 ± 9^*∗*^	86 ± 9	98 ± 10^*∗*^	83 ± 9^#^	85 ± 10^#^
	Female	81 ± 5	84 ± 7^@^	85 ± 7	97 ± 6^*∗*#^	78 ± 6^#^	88 ± 7^*∗*^
20	Male	86 ± 8	94 ± 8^*∗*^	90 ± 7	105 ± 10^*∗*#^	84 ± 8^#^	91 ± 8
	Female	82 ± 6	87 ± 6	89 ± 8	102 ± 9^*∗*#^	85 ± 7	93 ± 7^*∗*^
25	Male	94 ± 9	98 ± 9	96 ± 8	111 ± 9^*∗*#^	91 ± 10^#^	95 ± 7
	Female	87 ± 6	94 ± 7	98 ± 9^*∗*^	105 ± 10^*∗*#^	88 ± 9	94 ± 8
30	Male	99 ± 8	103 ± 10	111 ± 9^*∗*^	129 ± 11^*∗*#^	96 ± 8	102 ± 9
	Female	93 ± 7	95 ± 8	106 ± 8^*∗*#^	114 ± 11^*∗*#@^	95 ± 10	104 ± 9^*∗*#^
*Muscular TGs content (mg/g tissue)*							
5	Male	1.53 ± 0.12	1.52 ± 0.12	1.54 ± 0.10	1.6 ± 0.21	1.45 ± 0.11	1.5 ± 0.1
	Female	1.46 ± 0.12	1.53 ± 0.13	1.51 ± 0.14	1.55 ± 0.17^*∗*^	1.42 ± 0.13	1.48 ± 0.11
10	Male	1.75 ± 0.13	1.94 ± 0.14^*∗*^	1.81 ± 0.13	2.21 ± 0.2^*∗*#^	1.6 ± 0.12^*∗*#^	1.75 ± 0.15^#^
	Female	1.65 ± 0.13^@^	1.81 ± 0.14^*∗*^	1.66 ± 0.12^#@^	1.93 ± 0.16^*∗*@^	1.64 ± 0.14^#^	1.74 ± 0.13
15	Male	1.79 ± 0.12	2.11 ± 0.12^*∗*^	1.9 ± 0.14^#^	2.5 ± 0.22^*∗*#^	1.7 ± 0.16^#^	1.84 ± 0.14^#^
	Female	1.71 ± 0.13^@^	1.87 ± 0.13^*∗*@^	1.84 ± 0.14^*∗*^	2.28 ± 0.18^*∗*#@^	1.72 ± 0.17^#^	1.8 ± 0.13
20	Male	2.02 ± 0.11	2.17 ± 0.13^*∗*^	2.1 ± 0.12	2.7 ± 0.23^*∗*#^	2 ± 0.18^#^	2.1 ± 0.17
	Female	1.88 ± 0.13^@^	1.92 ± 0.14^@^	2.0 ± 0.17	2.43 ± 0.21^*∗*#@^	1.91 ± 0.14	1.98 ± 0.21
25	Male	2.14 ± 0.13	2.22 ± 0.16	2.3 ± 0.14^*∗*^	2.8 ± 0.21^*∗*#^	2 ± 0.12^#^	2.1 ± 0.18
	Female	1.97 ± 0.12^@^	2.13 ± 0.12	2.25 ± 0.16^*∗*#^	2.5 ± 0.18^*∗*#^	1.98 ± 0.19	2.11 ± 0.21^*∗*^
30	Male	1.89 ± 0.14	2.24 ± 0.15	2.3 ± 0.15^*∗*^	3 ± 0.26^*∗*#@^	2.1 ± 0.16^*∗*^	2.1 ± 0.17^*∗*^
	Female	1.84 ± 0.12	1.99 ± 0.13^@^	2.2 ± 0.16^*∗*#^	2.68 ± 0.21^*∗*#@^	2.07 ± 0.17^*∗*^	2.2 ± 0.12^*∗*#^

Data are presented as mean ± SD (*n* = 10). CF1-CD: F1 offspring of control mothers under control diet, CF1-HCD: F1 offspring of control mothers under HCD, OF1-CD: F1 offspring of obese mothers under control diet, and OF1-HCD: F1 offspring of obese mothers under HCD, MF1-CD: F1 offspring of malnourished mothers under control diet, and MF1-HCD: F1 offspring of malnourished mothers under HCD. The symbol *∗* indicates significant difference from CF1-CD by ANOVA (*p* < 0.05), the symbol # indicates significant difference from CF-HCD by ANOVA (*p* < 0.05), and the symbol @ indicates significant difference from male at each age by *t*-test.

**Table 2:** Serum NEFA level (mmol/mL) in different study groups.

Age (weeks)	Sex	CF1-CD	CF1-HCD	OF1-CD	OF1-HCD	MF1-CD	MF1-HCD
5	Male	1.14 ± 0.12	1.22 ± 0.13	1.2 ± 0.12	1.24 ± 0.15^*∗*^	1.10 ± 0.14	1.14 ± 0.13
	Female	1.22 ± 0.15	1.26 ± 0.14	1.3 ± 0.14	1.31 ± 0.13	1.24 ± 0.13^@^	1.3 ± 0.12^@^

10	Male	1.15 ± 0.12	1.33 ± 0.12^*∗*^	1.26 ± 0.10^*∗*^	1.38 ± 0.14^*∗*^	1.10 ± 0.12	1.33 ± 0.14^*∗*^
	Female	1.23 ± 0.12	1.37 ± 0.18^*∗*^	1.30 ± 0.11	1.45 ± 0.15^*∗*^	1.28 ± 0.14	1.32 ± 0.17

15	Male	1.21 ± 0.15	1.39 ± 0.14^*∗*^	1.32 ± 0.12	1.44 ± 0.12^*∗*^	1.16 ± 0.15	1.4 ± 0.16^*∗*^
	Female	1.30 ± 0.18	1.42 ± 0.13	1.34 ± 0.13	1.48 ± 0.16^*∗*^	1.32 ± 0.16	1.42 ± 0.15

20	Male	1.20 ± 0.11	1.42 ± 0.12^*∗*^	1.34 ± 0.11^*∗*^	1.5 ± 0.15^*∗*^	1.18 ± 0.15^#^	1.47 ± 0.16^*∗*^
	Female	1.33 ± 0.12	1.48 ± 0.12^*∗*^	1.35 ± 0.12	1.55 ± 0.13^*∗*^	1.34 ± 0.17^@^	1.41 ± 0.17

25	Male	1.19 ± 0.12	1.48 ± 0.13^*∗*^	1.37 ± 0.12^*∗*^	1.56 ± 0.14^*∗*^	1.11 ± 0.14^#^	1.52 ± 0.15^*∗*^
	Female	1.41 ± 0.13^@^	1.51 ± 0.12	1.40 ± 0.16	1.57 ± 0.13^*∗*^	1.35 ± 0.17^#@^	1.48 ± 0.19

30	Male	1.21 ± 0.15	1.53 ± 0.16^*∗*^	1.40 ± 0.10^*∗*^	1.64 ± 0.14^*∗*^	1.17 ± 0.11	1.62 ± 0.16^*∗*^
	Female	1.42 ± 0.21^@^	1.51 ± 0.15	1.44 ± 0.16	1.56 ± 0.16	1.36 ± 0.15^@^	1.62 ± 0.19^*∗*^

Data are presented as mean ± SD (*n* = 10). CF1-CD: F1 offspring of control mothers under control diet, CF1-HCD: F1 offspring of control mothers under HCD, OF1-CD: F1 offspring of obese mothers under control diet, and OF1-HCD: F1 offspring of obese mothers under HCD, MF1-CD: F1 offspring of malnourished mothers under control diet, and MF1-HCD: F1 offspring of malnourished mothers under HCD. The symbol *∗* indicates significant difference from CF1-CD by ANOVA (*p* < 0.05), the symbol # indicates significant difference from CF-HCD by ANOVA (*p* < 0.05), and the symbol @ indicates significant difference from male at each age by *t*-test.

**Table 3:** Serum levels of cytokines in different study groups.

Age (weeks)	Sex	CF1-CD	CF1-HCD	OF1-CD	OF1-HCD	MF1-CD	MF1-HCD
*Adiponectin (ng/mL)*							
5	Male	12.7 ± 1.1	12.6 ± 1.2	12.6 ± 1.3	12 ± 1.1	12.4 ± 1.3	12.3 ± 1.2
	Female	17.9 ± 1.5^@^	16.8 ± 1.7^@^	16.8 ± 1.9^@^	16 ± 1.5^*∗*@^	17.4 ± 1.6^@^	16.4 ± 2^*∗*@^
10	Male	13.3 ± 0.8	13.4 ± 1.3	12.6 ± 1.2	12.1 ± 1.2^*∗*^	12.9 ± 1.2	12.4 ± 1.3
	Female	17.8 ± 1.3^@^	16.7 ± 1.6^@^	16.1 ± 2^*∗*@^	16.1 ± 1.6^*∗*@^	18 ± 1.8^#@^	16.5 ± 1.6^@^
15	Male	13.7 ± 1.3	13.2 ± 1.3	13.0 ± 1.2	11.7 ± 1.1^*∗*#^	13.2 ± 1.4	12.6 ± 1.3
	Female	17.3 ± 1.6^@^	16.3 ± 1.2^@^	16.5 ± 2.1^@^	15.2 ± 2^*∗*@^	17.5 ± 2^@^	15.4 ± 2.2^*∗*@^
20	Male	14.2 ± 1.2	12.8 ± 1.3^*∗*^	12.8 ± 1.3^*∗*^	10.8 ± 1^*∗*#^	13.7 ± 1.3^*∗*#^	12.8 ± 1.4^*∗*^
	Female	16.9 ± 1.7^@^	16.7 ± 1.6^@^	16.3 ± 1.8^@^	15.1 ± 1.5^*∗*@^	17.5 ± 1.6^@^	15 ± 1.6^*∗*@^
25	Male	14.3 ± 1.2	12.3 ± 1.2^*∗*^	12.5 ± 1.2^*∗*^	10.4 ± 1.2^*∗*#^	13.9 ± 1.4^#^	12.6 ± 1.4^*∗*^
	Female	16.6 ± 2.1^@^	16.7 ± 2.2^@^	15.6 ± 2^@^	14.1 ± 1.4^*∗*#@^	18 ± 1.5^@^	14.8 ± 1.8^*∗*#@^
30	Male	14.9 ± 1.3	12.6 ± 1.3^*∗*^	11.5 ± 1.3^*∗*^	10 ± 1.1^*∗*#^	14 ± 1.5^#^	12.6 ± 1.3^*∗*^
	Female	16.7 ± 1.8^@^	16.5 ± 1.8^@^	15.1 ± 2.1^@^	13.4 ± 2^*∗*#@^	17 ± 2^@^	14.2 ± 1.2^*∗*#@^
*Leptin (ng/mL)*							
5	Male	0.52 ± 0.08	0.58 ± 0.08	0.6 ± 0.07	0.62 ± 0.1^*∗*^	0.6 ± 0.07	0.58 ± 0.06
	Female	1.7 ± 0.1^@^	1.64 ± 0.12^@^	1.74 ± 0.1^*∗*@^	1.82 ± 0.16^*∗*#@^	1.61 ± 0.18^@^	1.75 ± 0.15^*∗*@^
10	Male	0.4 ± 0.09	0.42 ± 0.09^*∗*^	0.4 ± 0.09^*∗*^	0.48 ± 0.03^*∗*^	0.35 ± 0.02^#^	0.38 ± 0.02^*∗*^
	Female	1.2 ± 0.1^@^	1.19 ± 0.13^@^	1.4 ± 0.15^*∗*#@^	1.57 ± 0.15^*∗*#@^	1.22 ± 0.17^@^	1.55 ± 0.17^*∗*#@^
15	Male	0.28 ± 0.1	0.44 ± 0.12^*∗*^	0.36 ± 0.04^*∗*#^	0.5 ± 0.04^*∗*#^	0.25 ± 0.02^#^	0.4 ± 0.03^*∗*^
	Female	0.91 ± 0.1^@^	0.97 ± 0.13^@^	1.1 ± 0.14^*∗*#@^	1.4 ± 0.18^*∗*#@^	1.1 ± 0.16^*∗*#@^	1.3 ± 0.2^*∗*#@^
20	Male	0.22 ± 0.12	0.43 ± 0.1^*∗*^	0.35 ± 0.03^*∗*^	0.48 ± 0.04^*∗*#^	0.25 ± 0.03^#^	0.42 ± 0.02^*∗*^
	Female	0.83 ± 0.11^@^	0.94 ± 0.08^@^	1.15 ± 0.16^*∗*#@^	1.3 ± 0.16^*∗*#@^	0.94 ± 0.1^*∗*@^	1.24 ± 0.2^*∗*#@^
25	Male	0.24 ± 0.1	0.43 ± 0.13^*∗*^	0.33 ± 0.02^*∗*^	0.47 ± 0.03^*∗*^	0.23 ± 0.02^#^	0.4 ± 0.03^*∗*^
	Female	0.84 ± 0.12^@^	0.94 ± 0.14^@^	1.1 ± 0.14^*∗*#@^	1.4 ± 0.12^*∗*#@^	0.94 ± 0.12^@^	1.2 ± 0.22^*∗*#@^
30	Male	0.2 ± 0.1	0.42 ± 0.12^*∗*^	0.31 ± 0.02^*∗*#^	0.43 ± 0.07^*∗*^	0.22 ± 0.01^#^	0.4 ± 0.03^*∗*^
	Female	0.9 ± 0.1^@^	0.95 ± 0.1^@^	1.1 ± 0.12^*∗*#@^	1.4 ± 0.12^*∗*#@^	0.99 ± 0.07^@^	1.3 ± 0.27^*∗*#@^
*TNF-α (pg/mL)*							
5	Male	18 ± 2.2	24 ± 2.1^*∗*^	24 ± 2.1^*∗*^	24 ± 2.3^*∗*^	18 ± 3^#^	19 ± 2.4^#^
	Female	23 ± 3.1^@^	23 ± 2.5	26 ± 2.5^*∗*^	30 ± 2.4^*∗*#@^	24 ± 2.1^@^	27 ± 2.2^*∗*#@^
10	Male	19 ± 2.4	23 ± 2.6^*∗*^	26 ± 2.4^*∗*#^	51 ± 4^*∗*#^	20 ± 2.3	32 ± 2.1^*∗*#^
	Female	26 ± 3.1^@^	26 ± 2.6^@^	31 ± 2.2^*∗*#@^	41 ± 3.8^*∗*#@^	28 ± 2.2^*∗*@^	34 ± 2.1^*∗*#@^
15	Male	18 ± 2	24 ± 2^*∗*^	29 ± 2	57 ± 5.3^*∗*#^	20 ± 3.4	37 ± 3^*∗*#^
	Female	28 ± 4^@^	32 ± 3^*∗*@^	36 ± 3.1^*∗*#@^	46 ± 3.4^*∗*#@^	29 ± 3.1^@^	42 ± 3.4^*∗*#@^
20	Male	21 ± 2.4	26 ± 2.3^*∗*^	32 ± 2.4^*∗*#^	63 ± 6^*∗*#^	24 ± 3.6^*∗*^	41 ± 3^*∗*#^
	Female	30 ± 3.5^@^	34 ± 2.7^*∗*@^	39 ± 3^*∗*#@^	56 ± 4.1^*∗*#@^	34 ± 2.7^*∗*@^	46 ± 3.3^*∗*#@^
25	Male	22 ± 2.3	28 ± 2.1^*∗*^	37 ± 2.1^*∗*#^	71 ± 5^*∗*#^	26 ± 3.7^*∗*^	46 ± 3.5^*∗*#^
	Female	30 ± 2^@^	35 ± 2.9^*∗*@^	41 ± 3.6^*∗*#@^	58 ± 4.5^*∗*#@^	36 ± 2.5^*∗*@^	48 ± 3.4^*∗*#^
30	Male	23 ± 2	32 ± 2.2^*∗*^	42 ± 2.2^*∗*#^	78 ± 7.2^*∗*#^	30 ± 3^*∗*^	55 ± 3.2^*∗*#^
	Female	33 ± 3.2^@^	35 ± 3.2^@^	46 ± 3.2^*∗*#@^	59 ± 4.3^*∗*#@^	39 ± 2.6^*∗*#@^	50 ± 4.1^*∗*#@^

Data are presented as mean ± SD (*n* = 10). CF1-CD: F1 offspring of control mothers under control diet, CF1-HCD: F1 offspring of control mothers under HCD, OF1-CD: F1 offspring of obese mothers under control diet, and OF1-HCD: F1 offspring of obese mothers under HCD, MF1-CD: F1 offspring of malnourished mothers under control diet, and MF1-HCD: F1 offspring of malnourished mothers under HCD. The symbol *∗* indicates significant difference from CF1-CD by ANOVA (*p* < 0.05), the symbol # indicates significant difference from CF-HCD by ANOVA (*p* < 0.05) and the symbol @ indicates significant difference from male at each age by *t*-test.
